# What Determines BIM Competition Results of Undergraduate Students in the Architecture, Engineering and Construction Industry?

**DOI:** 10.3390/bs12100360

**Published:** 2022-09-26

**Authors:** Yibin Ao, Panyu Peng, Jiayue Li, Mingyang Li, Homa Bahmani, Tong Wang

**Affiliations:** 1 College of Environment and Civil Engineering, Chengdu University of Technology, Chengdu 610059, China; 2 College of Management Science, Chengdu University of Technology, Chengdu 610059, China; 3 Department of Engineering Management, Sichuan College of Architectural Technology, Deyang 618014, China; 4 Faculty of Architecture and Built Environment, Delft University of Technology, 2628 CD Delft, The Netherlands

**Keywords:** BIM, technology acceptance model, theory of planned behavior, behavioral intention, competition results

## Abstract

Building Information Modeling (BIM) has become the development trend in the architecture, engineering, and construction (AEC) industry. BIM discipline competition is an effective way for students of AEC-related disciplines to integrate theory with practice, and it is a key link to cultivate qualified BIM practitioners. This study takes participants of the 8th National College BIM Graduation Design Innovation Competition as the research objects. The Technology Acceptance Model and the Theory of Planned Behavior are combined to build a driving factor model of the competitions. Contestants of the competition were asked to complete online questionnaires, and 451 valid samples were finally obtained. Structural Equation Modeling was used to fit the theoretical model, and it was found that: Behavioral Intention to use BIM is directly and positively affected by Attitude, Perceived Behavioral Control, and Perceived Usefulness, as well as indirectly and positively affected by Perceived Usefulness and Perceived Eased of Usefulness. Competition Results is directly and positively affected by Behavioral Intention to use BIM, Perceived Behavioral Control, and Facilitating Conditions. It is also indirectly and positively affected by Attitude, Perceived Behavior Control, Perceived Usefulness, and Perceived Eased of Usefulness. The results show that the situation of students’ participation in BIM competition can be optimized by increasing the publicity and promotion of BIM and related policies, strengthening the construction of supporting teaching facilities, building the integrated curriculum system of BIM technology, and strengthening teacher training. This study provides a theoretical reference for further BIM practice, and it would help improve the corresponding teaching organization and enhance the internal drive of students’ BIM learning.

## 1. Introduction

The construction industry has changed from traditional construction practice to BIM-based practice [1]. As the second revolution of the construction industry, BIM technology has effectively promoted the sustainable development of the construction industry [2]. However, most construction companies still do not have a holistic understanding of BIM [3], and there are still problems in the implementation of BIM technology. BIM’s implementation difficulties include poor government leadership, insufficient guidance, organizational problems, legal problems, high application costs, and insufficient external incentives [4]. The demand for BIM technical talents is urgent, and the development of the industry also forces colleges to offer BIM-related courses to cultivate BIM technical talents. In recent years, colleges and universities in China have successively set up BIM courses; however, the teaching effect is not ideal due to such bottlenecks as “the disconnect between teaching and practice”, “students’ lack of ability to solve problems independently”, “students’ lack of innovation practice”, “lack of experienced teachers”, and “poor hardware conditions” [5,6,7,8]. Colleges and universities must review the gap between BIM education and industry practice [1]. With the government’s strong support, some regions and enterprises have conducted various BIM competitions, hoping to stimulate students’ ability to connect theory with practice, which is lacking in the current BIM education [9,10]. However, more studies on the influencing factors of BIM competition and the effect of BIM competitions on the improvement of students’ comprehensive ability are needed to provide theoretical and empirical evidence to improve BIM competition and education.

Therefore, this study aims to understand the role of discipline competition in promoting students’ learning of BIM technology, software application, and mastery of professional knowledge. Taking the 8th National College BIM Graduation Design Innovation Competition as the platform, this study constructs the driving factors model based on the integrative application of the Technology Acceptance Model (TAM) and the Theory of Planned Behavior (TPB). Perceived Eased of Usefulness (PEU), Perceived Usefulness (PU), Attitude (AT), Subjective Norm (SN), Perceived Behavioral Control (PBC), Facilitating Conditions (FC), Behavioral Intention to use BIM (BI), and Competition Results (CR) are involved in the model to explore factors that affect the contestants’ competition results and their willingness to continue using BIM technology. This study intends to provide a theoretical basis for further improvement of the BIM discipline competition quality and BIM education quality.

## 2. Literature Review

### 2.1. Application of BIM

BIM is meant for the process of creating and managing information, which enables AEC professionals to effectively plan, design, construct, and manage the whole construction project’s life cycle [11]. With BIM’s implementation, project management’s complexity and difficulty will be significantly reduced [12], and productivity will be improved [3]. Many countries have been committed to promoting BIM technology. For example, since 2016, South Korea has required that all public buildings with a cost of more than USD 27.6 million must introduce BIM technology [4], and the British government formulated a strategic policy, expecting to achieve the complete collaboration of BIM in all public sector projects by 2016 [13]. However, the application of BIM has not reached the expected level. There are some problems in the practice process, such as insufficient understanding of BIM, lack of training, and ambiguous roles and responsibilities [13]. Howard et al. [13] found that current policies and research are mainly concentrated at the industry, enterprise, and project level, while the needs at the individual participant level have not gotten enough attention. Al-Ashmori et al. [3] found that most construction companies in Malaysia lack an understanding of BIM technology.

Compared with developed countries, the development of BIM technology in China has been initiated relatively late, but the policy support of the Chinese government for BIM technology has been continuous. In the “14th five-year plan for the development of housing and urban, rural construction science and technology”, the importance of BIM technology is emphasized again. The document mentioned to “study the theory, method, and support system of the integrated application of BIM technology and the new generation of information technology to support the digital transformation and development of the construction industry” [14]. Heng and Hong-yu [15] divided the research of BIM technology in China into two stages. The first stage is from 2002 to 2014 when scholars understand BIM in the primary stage. The second stage starts after 2015, during which BIM-related research is in the ascendant and has made some progress. Zhou et al. [4] divided the strategies prepared in China into five categories: BIM standards and guides, BIM software development, BIM training for talents, BIM-related academic research, and BIM best practices. With the vigorous promotion of the Chinese government, many construction projects have adopted BIM technology. However, there are still obstacles in construction projects, such as insufficient government guidance, insufficient external incentives, the unclear willingness of project participants to cooperate, a lack of professionals, and others [4,15,16]. At present, the adoption of BIM technology still lags behind expectations.

### 2.2. Teaching of BIM

The improvement of BIM education will positively impact the BIM awareness [2]. BIM modeling in architectural education is conducive to developing students’ project concepts and design ability [17]. Scholars have studied how to integrate BIM into the existing curriculum system and also studied problems in BIM education. Elgewely et al. [18] proposed to integrate BIM into architectural engineering education. They confirmed through empirical research that using Virtual Reality (V.R.) to realize students’ practical teaching can significantly improve their learning experience and performance. Clevenger et al. [19] studied the way to integrate BIM technology into architectural engineering-related courses. Casasayas et al. [20] divided reasons that hinder BIM education in Australian universities into four aspects: change management adjustment, curriculum and content restrictions, problems of educators, and disconnection from the industry. Through the research, they pointed out that researchers should provide the link between graduates’ preparation for BIM and their employability in the Australian market. They also argued that relevant research should highlight the industry’s needs. In addition, Puolitaival and Forsythe [21] proposed that the preparation and optimal use of practical models in high-quality education is a major challenge.

The training of BIM technical talents in China is still in infancy [16], and there are many problems, such as “lack of equipment and facilities”, “unsystematic curriculum setting”, “the teachers’ insufficient professionalism”, and “lack of systematic curriculum application” [22]. To improve the teaching effect of BIM technology, some colleges integrate BIM technology into the graduation design, hoping to strengthen the connection between BIM education and industry [23].

### 2.3. Discipline Competition

In order to make up for the problems of disconnection from actual projects and insufficient practical experience of students in BIM education, various competitions have been held in collaboration with industries, enterprises, and colleges. Due to a large number of practical engineering projects, the participation of enterprises in competitions can significantly improve the connection between students and practical engineering [24]. With the development of various competitions, scholars have conducted research on discipline competitions, mainly involving the pattern and problems of competitions, the combination of competition and school curriculum, and the impact on students’ personal ability [25,26,27,28,29,30]. Plakhotnik et al. [27] confirmed that the experience of participating in competitions significantly impacts the career decision-making and self-efficacy of participants. With the joint promotion of universities and enterprises, discipline competitions in China are booming. However, there are also some problems, such as “repeated content”, “limited project types”, and “a limited number of trainees” [26]. Currently, most of the research on discipline competition focuses on the innovative talent training mode and the operation status analysis. However, the internal influencing factors of students’ participation in discipline competitions have not been thoroughly studied. However, students are the main body of discipline competitions, so analyzing the internal influence in discipline competitions is necessary [28].

The existing studies are mainly carried out from the macro perspective, while more attention is needed at the personal level. The cultivation mode of BIM technical talents in colleges are still being explored, and there is no widely recognized system mode so far. Although the role of BIM discipline competitions in promoting BIM talent training has been demonstrated, there is less understanding of the competition’s effectiveness from the contestants’ perspective. Therefore, this study takes contestants as the object to study the influencing mechanism of the contestants’ competition results, expecting to provide a theoretical basis for improving BIM competition and BIM education.

## 3. Methodology

### 3.1. Research Theoretical Model

Davis et al. [31] proposed the TAM in 1989, which points out that the behavior of people facing new things will be affected by PEU, PU, AT, and BI [16]. Many scholars have used the TAM model to study the construction industry’s acceptance of BIM technology. Acquah et al. [32] used TAM to test the acceptance of BIM in Ghana’s construction industry, while Lee et al. [33] developed a BIM acceptance model based on the theory related to technology acceptance behavior. In addition, Zhao et al. [34] studied the specific mechanism of Chinese AEC professionals’ acceptance of BIM technology in combination with the TAM and Technology Organization Environment model.

Scholars also attempt to integrate various theoretical models to better explain people’s acceptance behavior in the face of new technologies. Venkatesh et al. [35] proposed the Unified Theory of Acceptance and Use of Technology (UTAUT) in 2003, which integrates eight main models: the Theory of Reasoned Action, the Technology Acceptance Model, the Motivation Model, the Theory of Planned Behavior, the combined TAM-TPB model, the Model of Personal Computer Utilization, the Innovation Diffusion Theory, and Social Cognition Theory.

In addition, TPB, as one of the most widely used theories in social and behavioral sciences, has been widely used in health science, environmental science, educational research, and other fields [36]. TPB mainly proposes that behavior intention is formed by three factors: attitude, subjective norms, and perceived behavior control. Some scholars also use TPB to explore the final use behavior of BIM technology. For example, Wu et al. [37] confirmed the applicability of the TPB with the study of critical factors of professionals’ BIM technology adoption behavior.

Generally, while participants have a firm behavior intention on BIM technology, they will have stronger motivation in the process of the competition to achieve better competition results. Howard et al. [13] verified the significant impact of behavioral intention on behavior in empirical research. When people are facing new technologies, their behavior depends on their attitude towards new technologies [16]. Botero et al. [38] confirmed that attitude is a powerful factor affecting behavioral intention. Zhou and Li [39] verified that the acceptability of secondary vocational students to the mixed teaching model would be affected by the people around them, while Venkatesh and Zhang [40] also verified that the influence of the surrounding environment and people can significantly impact individual behavior through comparing the social environment of China and the United States. In UTAUT, it is believed that the promotion conditions will also affect people’s final use behavior [40]. In addition, Ao et al. [1] pointed out that the results of students participating in competitions will be affected by the guidance of instructors. Therefore, the factor of “Facilitating Conditions” has also been considered in this study.

The variables in this study model are defined as follows.

CR: The final performance of a competitor in a BIM competition.
FC: How much support the individual feels from the organization for learning BIM technology and participating in the competition in terms of relevant technologies and equipment.
BI: Individual’s subjective judgment on the probability of continuing to learn BIM technology and engaging in relevant industries in the future.
AT: Individual’s positive or negative feelings about learning BIM technology and participating in the competition.
SN: The degree to which an individual perceives others around him as to whether he should learn BIM technology and enter the competition.
PU: The extent to which an individual believes learning BIM technology and participating in the competition will help his or her abilities or future career.
PBC: The extent to which individuals anticipate learning BIM technology and participating in the games that they can control or master.
PEU: How easy an individual thinks it is to learn BIM and compete.

Based on the above research conclusions and the team’s research basis for the BIM competition, this study puts forward the following assumptions:

**Hypothesis** **1** **(H1):** *AT of participants has a positive effect on BI;*

**Hypothesis** **2** **(H2):** *SN of participants has a positive effect on BI;*

**Hypothesis** **3** **(H3):** *PBC of participants has a positive effect on BI;*

**Hypothesis** **4** **(H4):** *BI has a positive impact on CR;*

**Hypothesis** **5** **(H5):** *PBC of participants has a positive effect on CR.*

When participants feel that learning BIM technology is not difficult and that BIM technology benefits the growth of their comprehensive personal ability, their attitude towards BIM technology may be more positive. Their intention to use BIM technology in the future may also be enhanced. Sun and Cao [16] verified that when students feel that learning BIM technology is helpful, they will learn BIM technology with a positive attitude, and their subsequent behavior will be affected. Meanwhile, when the difficulty of learning BIM technology is acceptable, students would think that learning and using BIM technology is useful, and their corresponding learning behavior would be promoted. Zhao et al. [34] also verified that perceived usefulness and perceived eased of usefulness have a significant positive effect on attitude. Therefore, this study proposes the following assumptions:

**Hypothesis** **6** **(H6):** *PU has a positive effect on AT;*

**Hypothesis** **7** **(H7):** *PEU has a positive effect on AT;*

**Hypothesis** **8** **(H8):** *PEU has a positive effect on PU;*

**Hypothesis** **9** **(H9):** *PU has a positive effect on BI.*

When participants can get adequate support, such as equipment and technical guidance, they would solve problems in a timely and effective manner in the competition, which may encourage them to achieve better competition results. Howard et al. [13] also confirmed that good external promotion conditions significantly positively impact the final use behavior. Therefore, the following assumptions are made:

**Hypothesis** **10** **(H10):** *FC has a positive effect on CR.*

**Figure 1:**
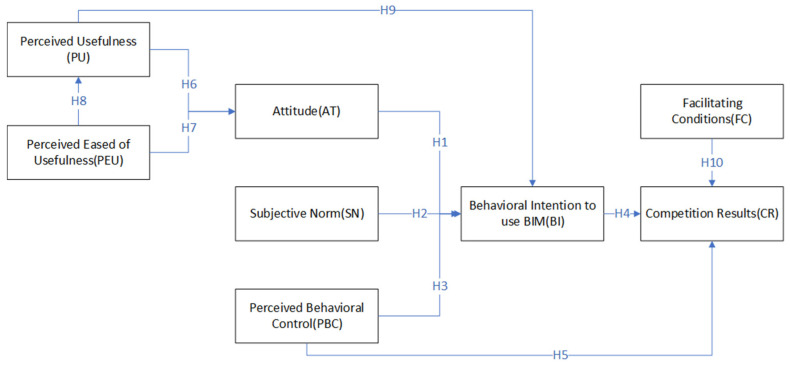
Driving factors model of BIM competition results based on TAM-TPB.

According to the above research assumptions, the systematic theoretical model proposed in this study is shown in Figure 1.

### 3.2. Data Collection

According to the above theoretical model, the questionnaire has been implemented to collect data. The questionnaire is mainly divided into two parts: the first part is the demographic variables of the contestants, including gender, grade, major, and others, and the second part is all the measurement items involved in the model. The five-point Likert scale was used to measure the degree of conformity between each observation variable and the contestant (1-completely disagree; 2-basically disagree; 3-average 4-basically agree; 5-completely agree). The specific items of the questionnaire are shown in Table 1.

The 8th National College BIM Graduation Design Innovation Competition, as one of the BIM competitions with high recognition, was held from October 2021 to May 2022. The competition provides a new pattern for the application exploration of BIM education and the curriculum of BIM-related courses. The competition includes nine aspects: civil construction BIM modeling and application, mechanical and electrical BIM modeling and application, the whole process cost management and application, BIM bidding management and application, BIM decoration design creativity and innovation, application of BIM construction project management, prefabricated building BIM design and construction, intelligent building and management innovation and BIM positive design application innovation. There are four prizes: first prize, second prize, third prize, and excellence award. More than 560 colleges and universities have joined the competition. This research is divided into two stages. The first stage was the pre-survey conducted from 28 March 2022 to 3 April 2022. After the pre-survey, the questionnaire is adjusted. The second stage is the formal investigation, conducted from 5 April to 28 May 2022. The questionnaire collection was completed with the assistance of the organizer, Glodon Company Limited, of the competition. The questionnaires were distributed to the participating students through the system during the competition. A total of 828 questionnaires were collected, of which 451 were valid, with an effective rate of 54.47%. Table 2 represents the interviewees’ statistics:

## 4. Results

### 4.1. Reliability and Validity

Cronbach’s α coefficient is used to test the reliability of the questionnaire. It is generally believed that the questionnaire has good reliability when the Cronbach’s α coefficient reaches 0.8 [37]. The overall Cronbach’s α coefficient of the questionnaire was 0.959. Additionally, the Cronbach’s α coefficient of PU, PEU, SN, FC, AT, PBC, and BI, respectively, are 0.948, 0.849, 0.930, 0.912, 0.915, 0.910, and 0.931, all of which reached above 0.8, indicating good reliability of the questionnaire.

Confirmatory factor analysis was used to test the validity of the questionnaire, and the test results showed that the KMO value was 0.947, which was higher than the critical value of 0.7 [44], and the *p*-value of significance was 0. Meanwhile, all factor loadings were between 0.629 and 0.924, which were at acceptable levels. The AVE was calculated to test the convergent validity of the questionnaire data. If the value was greater than 0.5, the test was passed; if the CR was greater than 0.7, the test was passed [45]. The test results are shown in Table 3, which shows that the results are good and can be further analyzed in the structural equation model.

### 4.2. Goodness of Fit

The fitting index of the structural equation model is shown in Table 4. All the indexes meet the recommended optimal value requirements except GFI. Although GFI does not meet the requirements of the optimal recommended value, it is also very close to the required 0.9, and it is also considered to be within the range of acceptability when GFI is greater than 0.8 [37], so it could be considered that the model has a good fitting. The model fitting results are shown in Table 4.

### 4.3. Path Analysis

**Figure 2:**
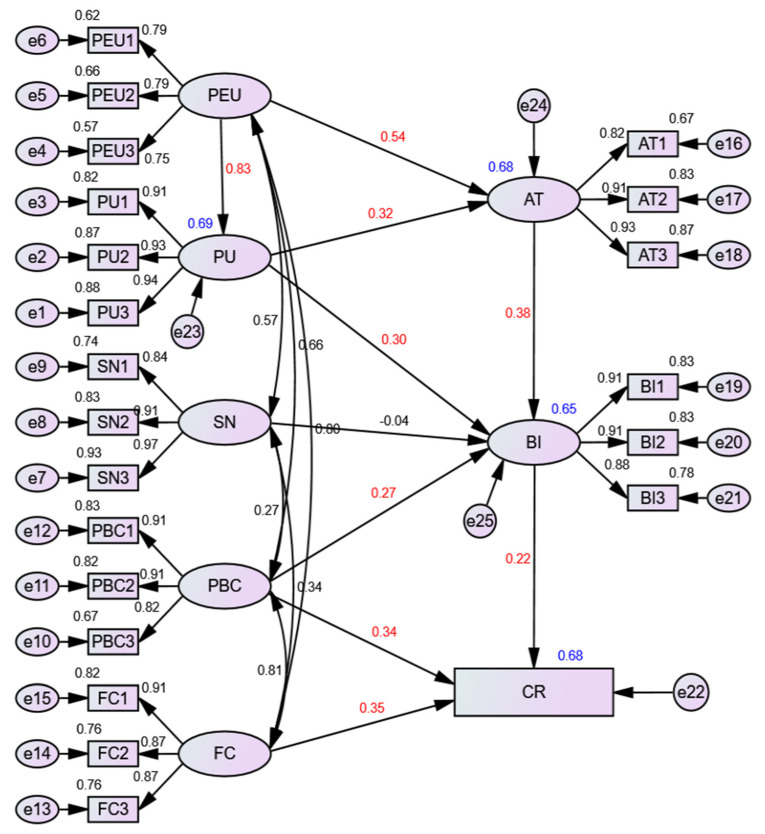
Model Analysis Diagram.

According to the empirical research results, except for hypothesis 2, all other hypotheses are accepted. The fitting results of the model are shown in Figure 2. The results of hypothesis testing are shown in Table 5.

### 4.4. Total Effect, Direct Effect, and Indirect Effect

**Figure 3:**
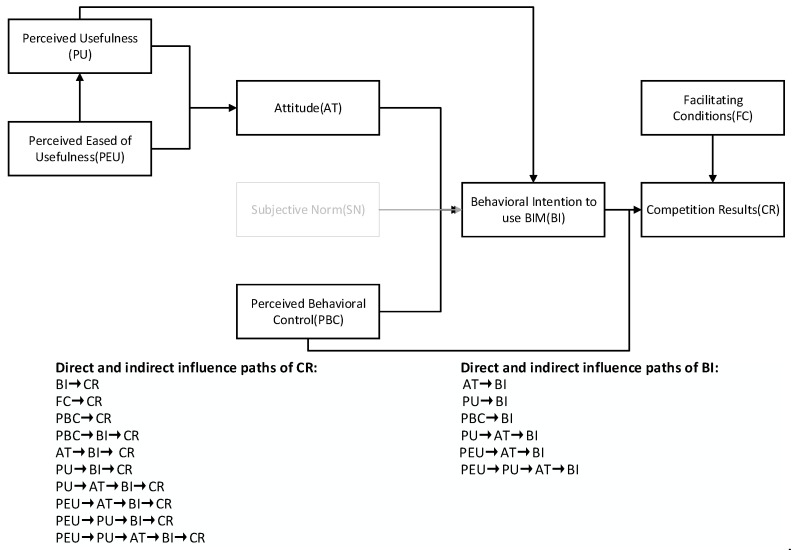
Relationship diagram between direct and indirect effect variables.

In order to clearly reflect the total effect, direct effect, and indirect effect among variables, the methods of “indirect, direct, and total effect” are used when exporting the results. The total effect, direct effect, and indirect effect among variables are shown in Table 6. The relationship between direct and indirect effect variables is shown in Figure 3.

## 5. Discussion

PEU has a significant positive impact on CR (0.122). As can be seen from Table 6 and Figure 2, the action path of PEU on CR can be divided into three aspects: PEU → AT → BI → CR, PEU → PU → AT → BI → CR, PEU → PU → BI → CR. PEU had directly significant positive effects on AT (0.541) and PU (0.834). Meanwhile, PEU has an indirectly significant positive effect on AT (0.267) through “PU → AT (0.321)”, which strengthens the total effect of PEU on AT (0.808). Similarly, PEU has an indirectly significant positive impact on the BI (0.553) through “AT→ BI (0.377)” and “PU→ BI (0.298)”. Finally, PEU has an indirectly significant positive impact on CR (0.122) through “BI→ CR (0.221)” (PEU has no direct effect on competition performance, but there is indirect effect: PEU → AT → BI → CR = 0.541 × 0.377 × 0.221 = 0.045; PEU → PU → AT → BI → CR = 0.834 × 0.321 × 0.377 × 0.221 = 0.022; PEU → PU → BI → CR = 0.034 × 0.298 × 0.221 = 0.055; indirect effect = 0.045 + 0.022 + 0.056 = 0.122; total effect = direct effect + indirect effect = 0 + 0.122 = 0.122; the calculation process of indirect effect and total effect of other paths are consistent with this). Davis [46] believes that individual experience in using new technologies significantly impacts PEU and PU While technology is simple to use, its usefulness will be improved accordingly. Notably, the total effect of PEU and PU on BI is ranked first and second. Sun and Cao [16] believe that when college students feel that learning BIM technology is useful, they will take a positive attitude toward learning BIM technology. Zhao et al. [34] verified that professionals’ PEU and PU of BIM technology would significantly affect their attitude towards BIM technology. So, improving PEU and PU is an effective way to promote the willingness to use BIM technology and can further effectively improve students’ performance in BIM competition. Therefore, in the teaching process of AEC majors, BIM modeling courses should be implanted earlier so that students can understand BIM technology earlier and realize the benefits of BIM technology to improve their professional ability and their acceptance of BIM technology. Moreover, students’ PEU and PU of BIM technology should be improved through more practical operation links.

PBC has a significant positive impact on CR (0.404), and there are two influencing paths: PBC → CR, PBC → BI → CR. PBC has a directly and positively significant impact on BI (0.273) and CR (0.344). At the same time, the PBC has an indirectly and positively significant effect on CR (0.060) through “BI → CR (0.221)”, which strengthens the total effect of PBC on CR. In conclusion, the overall effect of PBC on CR is the largest. This indicates that the stronger the confidence and belief of participants in completing the competition, the stronger the willingness to continue to learn BIM technology and engage in relevant positions in the future, which can indirectly improve the competitive performance of participants. Shi et al. [28] believe that lack of confidence may lead students to give up participating in the competition, which is consistent with the results of this study. Meanwhile, if the participants have reasonable confidence and belief, they will keep a positive attitude in the competition process and learn BIM technology more actively. After an in-depth understanding of BIM technology, their willingness to use it in the future will be promoted [13]. According to the result, improving PBC is an effective way to improve CR First, colleges should help students lay a good foundation in professional courses so that students could get familiar with the basic concept of BIM technology and software operation, which might help them to reduce the difficulty of participating in the competition. Second, colleges and enterprises can give students more opportunities to exercise their comprehensive ability to improve their self-confidence and give full play to their initiative in learning. This tool may help to improve the PBC of students.

FC, the second major factor affecting the CR, has a directly and positively significant impact on CR (0.350). Shi et al. [28] pointed out that students are easy to be influenced by external factors, such as lack of competition information and inactive instructors. These influences hinder students from participating in competitions. Howard et al. [13] also verified the positive relationship between external promoting conditions and students’ personal use of BIM technology. It is consistent with the results of this study. This conclusion also reminds colleges to increase investment in BIM education and provide more suitable conditions to support students in learning BIM technology.

SN does not significantly influence BI, so there is no significant indirect influence on CR through “BI → CR”. This result is different from the research conclusions of Howard et al. [13] and Wu et al. [37], whose studies verified that individuals would be affected by surrounding people. The reason may be that their research is aimed at industry employees, and whether industry employees adopt BIM technology is more subject to the requirements of enterprises and industries [13]. In contrast, the participants in this study are students, and their feelings and gains in the competition are more based on individual feelings. As a result, students are less affected by their surroundings.

## 6. Conclusions

### 6.1. Conclusions and Suggestion

In this study, participants of the 8th National BIM Graduation Design Innovation Competition were selected as the objects to explore factors that influence participants’ performance and willingness to continue using BIM technology. A TAM-TPB-based driving factors model of the competition was built from the perspective of PEU, PU, AT, SN, PBC, FC, BI, and CR. The following conclusions are drawn through this study:

(1) CR is directly and positively affected by BI, PBC, and FC, while it is indirectly and positively affected by AT, PBC, PU, and PEU. In addition, PBC has the most considerable total effect on CR.
(2) BI is positively and directly significantly affected by AT, PBC, and PU, as well as positively and indirectly significantly affected by PU and PEU. Moreover, PEU has the largest total effect on BI.

To help ACE major students master BIM technology and complete the transition between school and industry, the following suggestions, which are combined with the current situation of BIM education, are put forward:

(1) Increasing the publicity and promotion of BIM and related policies: In the current situation, students lack interest and initiative in BIM learning because of problems such as inadequate understanding. This study suggests that schools or enterprises carry out related lectures, activities, and competitions to preach BIM technology and relevant policies, and promote the development of BIM education through the “FC→ CR” path. Moreover, professional course teachers can also have positive guidance for BIM technology to promote students’ perceived usefulness. So that students would have a positive attitude to BIM technology and achieve good results in BIM competitions through the paths of “PU → AT → BI → CR” and “PU → BI → CR”.
(2) Strengthening the construction of supporting teaching facilities: At present, the supporting facilities and training sites of BIM teaching are not enough to support students in carrying out practical training. This survey shows that PEU has the largest total effect on BI, so it is important to improve students’ PEU. Appropriate hardware and software support can help students learn BIM technology, thus improving academic PEU and helping to complete the competition. So, colleges should create good conditions, such as increasing the investment in teaching software and hardware, deepening school-enterprise cooperation, improving the basic practice teaching platform, building a special BIM training room, and completing BIM teaching software, for BIM teaching to motivate students to learn BIM and participate in competitions.
(3) Building the integrated curriculum system of BIM technology: To improve the connection between professional courses at different stages, it is suggested to use the same project to teach in different courses. For example, use the same project to carry out teaching in courses such as architectural engineering drawing, architectural engineering budget estimate, REVIT, and others [19]. Multi-stage training of the same project can effectively improve students’ PEU and confidence in BIM learning and competition, and it could effectively improve competition performance and practical ability of students through the paths of “PEU → AT → BI → CR”.
(4) Strengthening the training of teachers: At present, most college teachers lack engineering practice experience. Therefore, colleges should organize industry lectures, training, and other activities. This pattern could help to strengthen the school-enterprise linkage and enrich the practical experience of teachers. People with rich practical experience in the industry can also be hired as guest lecturers to teach in case of insufficient teachers [5]. Teachers with rich professional experience can effectively promote students’ learning of BIM technology and their PEU, and thus realize a virtuous circle of “PEU → AT → BI → CR” path. In addition, they will provide professional guidance to contestants and improve students’ performance in the competition through the “FC → CR” path.

### 6.2. Deficiencies and Further Research

Although the number of questionnaires is enough to complete the study, the questionnaire recovery rate is not ideal, which needs to be improved in future research. Moreover, the questionnaire was completed at one time. Participants completed the questionnaire according to their feelings during the whole process of the competition, and the process of attitude change during the competition was not taken into account.

In the following research, participants will be longitudinally tracked in different stages of the competition so that their competition situation and attitude change will be understood according to the tasks and progress of each stage. At the same time, the career development of participants who have participated in the competition would be followed up to understand their attitude changes after entering the workplace to provide a theoretical basis for improving the effect of BIM education and also help to complete the connection between school education and vocational positions.

## Figures and Tables

**Table 1:** Measuring Scale.

Latent Variables	Codes	Observed Variables	Source
PEU	PEU1	Participating in BIM competition can improve professional knowledge.	[31,34,35]
	PEU2	Participating in BIM competition can improve team cooperation ability and communication ability.	
	PEU3	Learning BIM technology is helpful for my career development.	
PU	PU1	BIM competition is not difficult for me.	[31,34,35]
	PU2	The interface design of BIM software is reasonable and easy to operate.	
	PU3	BIM software is easy to learn and easy to use.	
SN	SN1	People who affect my behavior think I should participate in the BIM competition.	[35,36,37,41,42]
	SN2	People who are important to me think I should participate in the BIM competition.	
	SN3	People who affect my behavior think I should learn BIM technology.	
PBC	PBC1	Even if there is no help, I am confident to complete the BIM graduation design competition.	[35,36,37,41,42]
	PBC2	Even if I have no BIM competition experience before, I am confident to complete the BIM graduation design innovation competition.	
	PBC3	As long as I try my best, I can always solve problems encountered in the BIM competition.	
FC	FC1	When I encounter difficulties during the competition, instructors/classmates can help me solve difficulties.	[1,35,40]
	FC2	BIM technology is related to my major.	
	FC3	My school supports us in participating in BIM competitions.	
AT	AT1	Learning BIM technology is interesting.	[13,16]
	AT2	It’s fun to participate in the BIM competition.	
	AT3	I like to participate in the BIM competition.	
BI	BI1	I intend to continue to study/use BIM technology.	[27,43]
	BI2	If I am engaged in the construction industry in the future, I will give priority to BIM-related positions.	
	BI3	I would pay more attention to BIM-related positions while looking for part-time internships and employment.	

**Table 2:** Demographic distribution.

Characteristics		Sample	
		Frequency	Proportion (%)
Gender	Male	251	55.7
	Female	200	44.3
Age	Under 19	9	2.0
	19–21	256	56.8
	22–24	179	39.7
	25 and above	7	1.5
Major	Engineering management	125	27.7
	Engineering cost	146	32.4
	Civil engineering	100	22.2
	Water supply and drainage science and engineering	7	1.6
	Building environment and energy application engineering	10	2.2
	Building electrical and intelligent	8	1.8
	Other	55	12.2
Grade	Freshman	13	2.9
	Sophomore	116	25.7
	Junior	197	43.7
	Senior	125	27.7

**Table 3:** Test Results of the Measurement Model.

Variable	CR	AVE	Distinctions						
			PU	PEU	SN	PBC	FC	AT	BI
PU	0.949	0.861	0.928						
PEU	0.8533	0.659	0.775	0.812					
SN	0.932	0.822	0.518	0.561	0.906				
PBC	0.912	0.776	0.578	0.572	0.292	0.881			
FC	0.913	0.778	0.708	0.731	0.366	0.799	0.882		
AT	0.919	0.791	0.771	0.724	0.371	0.728	0.767	0.889	
BI	0.931	0.819	0.709	0.719	0.335	0.664	0.796	0.763	0.905

**Table 4:** Structural model fitting index.

Index	CMIN/DF	GFI	AGFI	RMSEA	NFI	RFI	IFI	TLI	CFI
Text Value	2.878	0.896	0.864	0.065	0.944	0.934	0.963	0.956	0.963
Recommended Value	1~3	>0.9	>0.9	<0.08	>0.9	>0.9	>0.9	>0.9	>0.9
Source	[34,37]								

**Table 5:** Hypothesis test results.

Path	Hypothesis	Std.	S.E	C.R.	*p*	Verified Results
BI <— AT	H1	0.377	0.077	5.634	***	Accepted
BI <— SN	H2	−0.043	0.052	−1.111	0.352	Rejected
BI <— PBC	H3	0.273	0.058	5.566	***	Accepted
CR <— BI	H4	0.211	0.048	4.622	***	Accepted
CR <— PBC	H5	0.344	0.068	5.946	***	Accepted
AT <— PU	H6	0.321	0.077	4.279	**	Accepted
AT <— PEU	H7	0.541	0.098	6.514	***	Accepted
PU <— PEU	H8	0.834	0.057	16.795	***	Accepted
BI <— PU	H9	0.298	0.072	4.935	***	Accepted
CR <— FC	H10	0.35	0.079	5.232	***	Accepted

Note: *** means *p* < 0.001, ** means *p* < 0.01.

**Table 6:** Total effect, direct effect and indirect effect.

		FC	PBC	SN	PEU	PU	AT	BI
**PU**	Total	—	—	—	0.834 ***	—	—	—
	Direct	—	—	—	0.834 ***	—	—	—
	Indirect	—	—	—	—	—	—	—
**AT**	Total	—	—	—	0.808 ***	0.321 **	—	—
	Direct	—	—	—	0.541 ***	0.321 **	—	—
	Indirect	—	—	—	0.267 **	—	—	—
**BI**	Total	—	0.273 ***	−0.043	0.553 ***	0.419 ***	0.377 ***	—
	Direct	—	0.273 ***	−0.043	—	0.298 ***	0.377 ***	—
	Indirect	—	—	—	0.553 ***	0.121 **	—	—
**CR**	Total	0.350 ***	0.404 ***	−0.009	0.122 ***	0.093 ***	0.083 ***	0.221 ***
	Direct	0.350 ***	0.344 ***	—	—	—	—	0.221 ***
	Indirect	—	0.060 ***	−0.009	0.122 ***	0.093 ***	0.083 ***	—

Note: *** means *p* < 0.001, ** means *p* < 0.01.

## Data Availability

The data used to support the findings of this study are available from the corresponding author upon reasonable request.

## References

[1] Ao Y., Liu Y., Tan L., Tan L., Zhang M., Feng Q., Zhong J., Wang Y., Zhao L., Martek I. (2021). Factors Driving BIM Learning Performance: Research on China’s Sixth National BIM Graduation Design Innovation Competition of Colleges and Universities. Buildings.

[2] Atabay S., Pelin Gurgun A., Koc K. (2020). Incorporating BIM and Green Building in Engineering Education: Assessment of a School Building for LEED Certification. Pract. Period. Struct. Des. Constr..

[3] Al-Ashmori Y.Y., Othman I., Rahmawati Y., Amran Y.H.M., Sabah S.H.A., Rafindadi A.D.u., Mikić M. (2020). BIM benefits and its influence on the BIM implementation in Malaysia. Ain Shams Eng. J..

[4] Zhou Y., Yang Y., Yang J.-B. (2019). Barriers to BIM implementation strategies in China. Eng. Constr. Archit. Manag..

[5] Fu Y. (2021). Research on the Training Mode of BIM Application Talents in Higher Vocational Colleges Based on CDIO Concept. Qual. Mark..

[6] Huang D. (2019). Exploration of BIM Course Teaching Reform Based on CDIO Concept and TBL Model. China Hous. Facil..

[7] Wu X., Chen R. (2021). Teaching Mode Reform and Application of ‘BIM Technology Application’ Course. Creat. Living.

[8] Ye J., Qian D., Yu X. (2021). Analysis of BIM Technology in Higher Vocational Civil Engineering Majors—Taking Bozhou Vocational and Technical College as an example. China South. Agric. Mach..

[9] Chen S., Chen M. (2020). Research on the influencing factors of college students ‘participation in subject competition. Econ. Outlook Bohai Sea.

[10] Wang L., Yin H. (2021). The Development and Application of BIM Technology for University Students in Professional Competition. Refrig. Air Cond..

[11] Autodesk What Is BIM, Building Information Modeling. https://www.autodesk.eu/solutions/bim.

[12] He Q., Wang G., Luo L., Shi Q., Xie J., Meng X. (2017). Mapping the managerial areas of Building Information Modeling (BIM) using scientometric analysis. Int. J. Proj. Manag..

[13] Howard R., Restrepo L., Chang C.-Y. (2017). Addressing individual perceptions: An application of the unified theory of acceptance and use of technology to building information modelling. Int. J. Proj. Manag..

[14] Ministry of Housing and Urban-Rural Development of the People’s Republic of China Science and Technology Development Plan of Housing and Urban-Rural Construction in the 14th Five-Year Plan. https://www.mohurd.gov.cn/gongkai/fdzdgknr/zfhcxjsbwj/202203/20220311_765107.html.

[15] Heng L., Hong-yu L. (2020). Applications of BIM in Construction Engineering in China: A Review. E3S Web Conf..

[16] Sun Y., Cao S. (2021). Factors of willingness to learn BIM technology in college students based on technology acceptance model. Proj. Manag. Technol..

[17] SoydaŞ ÇAkir H., Uzun T. (2020). Building Information Modelling in Architectural Education: Contribution of Bim in Design Process. Turk. Online J. Des. Art Commun..

[18] Elgewely M.H., Nadim W., ElKassed A., Yehiah M., Talaat M.A., Abdennadher S. (2021). Immersive construction detailing education: Building information modeling (BIM)–based virtual reality (VR). Open House Int..

[19] Clevenger C., Glick S.G., Puerto C.L.D. (2007). Interoperable Learning Leveraging Building Information Modeling (BIM) in Construction Education. Int. J. Constr. Educ. Res..

[20] Casasayas O., Hosseini M.R., Edwards D.J., Shuchi S., Chowdhury M. (2021). Integrating BIM in Higher Education Programs: Barriers and Remedial Solutions in Australia. J. Archit. Eng..

[21] Puolitaival T., Forsythe P. (2016). Practical challenges of BIM education. Struct. Surv..

[22] Jia X. (2021). Research and Education Status Analysis of Building Information Technology in China. Real Estate World.

[23] Li J. (2021). Discussion on Graduation Design Reform of Engineering Management Specialty Based on BIM. Sci. Technol. Inf..

[24] Jiang J. (2020). Research on BIM Second Classroom Teaching Mode Driven by Subject Competition. Educ. Mod..

[25] Fu L. (2018). Discussion on Higher Vocational Colleges Relying on Vocational Skills Competition to Improve the Quality of Personnel Training. Co-Oper. Econ. Sci..

[26] Lu G., Chen L., He Q., Yan H. (2018). The Evaluation of Academic Competition in Universities:Plan, Method and Exploration. China High. Educ. Res..

[27] Plakhotnik M.S., Krylova A.V., Maslikova A.D. (2020). Does participation in case competitions improve career decision-making self-efficacy of university students?. Educ. + Train..

[28] Shi M., Shi L., Ge F. (2019). Factors Influencing College Students Participating in Discipline Competition Based on Questionnaire Survey. J. Hubei Univ. Educ..

[29] Wang S. (2018). The applied undergraduate civil engineering major is based on BIM practica teaching research of design competition mechanism. Inf. Rec. Mater..

[30] Yang Y., Ha M. (2018). Study on the influence factors of teaching quality of BIM in the mode of promoting teaching and learning by competition. J. Hebei Univ. Eng. (Nat. Sci. Ed.).

[31] Davis F.D., Bagozzi R.P., Warshaw P.R. (1989). User Acceptance of Computer Technology: A Comparison of Two Theoretical Models. Manag. Sci..

[32] Acquah R., Eyiah A.K., Oteng D. (2018). Acceptance of Building Information Modelling: A survey of professionals in the construction industry in Ghana. J. Inf. Technol. Constr. (ITcon).

[33] Lee S., Yu J., Jeong D. (2015). BIM Acceptance Model in Construction Organizations. J. Manag. Eng..

[34] Zhao Y., Sun Y., Zhou Q., Cui C., Liu Y. (2022). How AEC professionals accept BIM technologies in China: A technology acceptance model perspective. Eng. Constr. Archit. Manag..

[35] Venkatesh V., Morris M.G., Davis G.B., Davis F.D. (2003). User Acceptance of Information Technology: Toward a Unified View. MIS Q..

[36] Bosnjak M., Ajzen I., Schmidt P. (2020). The Theory of Planned Behavior: Selected Recent Advances and Applications. Eur. J. Psychol..

[37] Wu Z., Jiang M., Li H., Luo X., Li X. (2021). Investigating the Critical Factors of Professionals’ BIM Adoption Behavior Based on the Theory of Planned Behavior. Int. J. Env. Res Public Health.

[38] Botero G.G., Questier F., Cincinnato S., He T., Zhu C. (2018). Acceptance and usage of mobile assisted language learning by higher education students. J. Comput. High. Educ..

[39] Zhou P., Li B. (2022). Influencing Factors of Secondary Vocational Students Acceptance of Blended Learning: Based on the Modified UTAUT Mode. J. Zaozhuang Univ..

[40] Venkatesh V., Zhang X. (2014). Unified Theory of Acceptance and Use of Technology: U.S. Vs. China. J. Glob. Inf. Technol. Manag..

[41] Ajzen I. (1991). The Theory of Planned Behavior. Organ. Behav. Hum. Decis. Processes.

[42] Neto I.L., Matsunaga L.H., Machado C.C., Günther H., Hillesheim D., Pimentel C.E., Vargas J.C., D’Orsi E. (2020). Psychological determinants of walking in a Brazilian sample: An application of the Theory of Planned Behavior. Transp. Res. Part F Traffic Psychol. Behav..

[43] Fishbein M., Ajzen I. (1975). Belief, Attitude, Intention, and Behavior: An Introduction to Theory and Research.

[44] Wang Z., Guo D., Wang X., Zhang B., Wang B. (2018). How does information publicity influence residents’ behaviour intentions around e-waste recycling?. Resour. Conserv. Recycl..

[45] Fornell C., Larcker D.F. (1981). Evaluating Structural Equation Models with Unobservable Variables and Measurement Error. J. Mark. Res..

[46] Davis F.D. (1989). Perceived usefulness, perceived ease of use, and user acceptance of information technology. MIS Q..

